# Floor-to-stand transition strategies observed in individuals with unilateral transfemoral amputation

**DOI:** 10.3389/fresc.2026.1959397

**Published:** 2026-10-23

**Authors:** Yukihiko Mizuno, Ryota Morishima, Shio Katoh, Genki Hisano, Hiroaki Hobara

**Affiliations:** 1 Department of Materials Science and Technology, Graduate School of Advanced Engineering, Tokyo University of Science, Tokyo, Japan; 2 Prosthetics and Orthotics Support Center, Tetsudo Kosaikai Foundation, Tokyo, Japan; 3 Arts et Metiers Institute of Technology, EPF Engineering School, Université Sorbonne Paris Nord, IBHGC-Institut de Biomécanique Humaine Georges Charpak, Paris, France; 4 Department of Medical and Robotic Engineering Design, Faculty of Advanced Engineering, Tokyo University of Science, Tokyo, Japan

**Keywords:** above-knee prosthesis, amputee, floor activity, floor-to-stand, kinematics

## Abstract

Lower-limb amputation is frequently associated with impaired mobility and an elevated risk of falls, making the restoration of functional independence a primary objective in rehabilitation. The ability to rise from the floor—referred to as the floor-to-stand transition (FTST)—is essential for post-fall recovery; however, the movement strategies employed by individuals with unilateral transfemoral amputation (uTFA) remain poorly characterized. This study aimed to characterize FTST strategies in a sample of individuals with uTFA and examine potential demographic differences between the observed strategy groups. Sixteen individuals with uTFA performed a single FTST trial from a long-sitting position. Movements were video-recorded and evaluated by independent reviewers. Two distinct FTST strategies were observed in the present sample: (i) a rise primarily supported by the intact limb with assistance from one or both upper limbs (*n* = 11), and (ii) a trunk rotation into a quadruped-like posture followed by bilateral hand support to achieve standing (*n* = 5). No statistically significant differences in demographic or clinical characteristics were observed between groups. However, participants who adopted the trunk-rotation strategy tended to be older and required more time to complete the FTST than those who adopted the intact-limb strategy, although these differences did not reach statistical significance. These findings highlight the compensatory biomechanical adaptations employed by individuals with uTFA in the absence of active knee control. Rehabilitation should therefore emphasize movement-specific training that accounts for biomechanical demands and age-related differences to enhance safety and efficiency during post-fall recovery.

## 1 Introduction

Lower-limb amputation (LLA) is frequently associated with substantial impairments in mobility, and the restoration of ambulatory function remains a primary objective in post-amputation rehabilitation. Previous studies have demonstrated that individuals with major LLA who achieve higher levels of mobility report superior quality of life (QoL) and greater life satisfaction compared to those with lower mobility outcomes (1). Despite these benefits, individuals with LLA exhibit a markedly elevated risk of falling. Epidemiological evidence indicates that between more than half of individuals with LLA experience at least one fall annually (2, 3). Accordingly, previous works have focused on who is at elevated risk (4–6), where falls occur (7), and which reactive strategies are used to prevent a complete fall (8–10). However, how people with LLA get back on their feet once they have fallen remains unclear, and systematic descriptions of post-fall floor-to-stand strategies are still limited.

The ability to rise independently from the floor—commonly referred to as the floor-to-stand transition (FTST)—is essential for regaining autonomy following a fall. Notably, it has been reported that approximately 53% of individuals with LLA were unable to perform the FTST without assistance (11), indicating the significant physical limitations experienced by this population. In addition, a previous study has shown that nearly half of individuals with transtibial amputation (TTA) utilize a half-kneeling strategy to rise from the floor (12). However, little is known about the movement strategies employed by individuals with unilateral transfemoral amputation (uTFA), who face unique biomechanical challenges due to the absence of a biological knee joint and increased reliance on prosthetic components.

Despite the critical role of FTST in post-fall recovery and rehabilitation, movement strategies adopted by individuals with uTFA remain insufficiently investigated. While some functional assessment tools include tasks related to rising from a seated or supine position, these components are typically embedded within broader multidimensional indices and lack detailed kinematic evaluation. For instance, Iwase et al. (2011) used a video-based classification system to identify FTST strategies in older adults. They identified three distinct strategies for transitioning from the supine position to standing: (i) rising from a position in which both hands and feet are in contact with the floor while both legs are extended, (ii) rising through a half-kneeling position, and (iii) rising through a squatting position (13). The authors also reported an association between FTST strategy and body mass index (BMI). However, such detailed movement-level analyses have rarely been conducted in individuals with lower-limb amputation. Moreover, systematic reviews have shown that in various healthy population, FTST strategies are known to differ depending on age and physical characteristics such as BMI. These findings suggest that individuals with uTFA may also exhibit multiple distinct FTST patterns, potentially classified according to demographic characteristics (14). However, the specific strategies used by individuals with uTFA during FTST remain unclear, and it is unknown whether distinct subgroups exist based on these movement patterns.

A more comprehensive understanding of FTST strategies employed by individuals with uTFA may inform the development of targeted rehabilitation interventions aimed at enhancing mobility, safety, and functional independence. Accordingly, this study aimed to characterize FTST strategies in a sample of individuals with uTFA/KD and examine potential demographic differences between the observed strategy groups. It was hypothesized that: (i) at least two distinct FTST strategies would be identified within the uTFA cohort, and (ii) these strategy groups would exhibit significant differences in key demographic variables.

## 2 Method

### 2.1 Participants

Participants were recruited through collaborating certified prosthetists using a targeted recruitment approach. The prosthetists were asked to identify potential participants who met the predefined eligibility criteria, including the ability to safely and independently perform the FTST. Because potential participants were initially identified by the collaborating prosthetists before referral to the research team, the total numbers of individuals considered for participation, those unable to perform the FTST independently, and those who declined participation were not systematically recorded by the research team.

Sixteen participants with uTFA (10 males, 6 females; age [mean ± standard deviation], 48.8 ± 14.5 years; height, 1.63 ± 0.10 m; mass, 59.5 ± 12.4 kg) participated in this study (Table 1). Fifteen participants had a unilateral transfemoral amputation and one had a unilateral knee disarticulation. The inclusion criteria were (1) unilateral transfemoral amputation or knee disarticulation, as confirmed by the participant's medical records, (2) no neuromuscular disorders or complications, (3) an age of 18 years or older at the time of participation, and (4) the ability to safely and independently perform a floor-to-stand transition.

**Table 1:** Participant’ s demographic data. *Note that the definitions of residual limb length are as follows: Short (≤1/3), Middle (>1/3 and <2/3), Long (≥2/3), KD (Knee Disarticulation).

Sex	Age (year)	Height (m)	Mass (kg)	BMI (kg/m*2)	TSAmp (year)	Amputation Side	Cause of Amputation	Residual Limb Length	Prosthetic Knee Unit	Experimental site
M	31	1.73	64.5	21.5	11	Right	Non-Trauma	Medium	NMPK	I
M	34	1.70	68.4	23.7	25	Right	Non-Trauma	Long	MPK	I
M	34	1.65	73.9	27.1	13	Right	Trauma	Medium	MPK	I
F	48	1.48	45.1	20.6	25	Right	Non-Trauma	Long	MPK	O
M	62	1.70	65.2	22.6	39	Left	Trauma	KD	MPK	O
M	64	1.69	54.5	19.1	13	Left	Non-Trauma	Long	NMPK	O
M	50	1.66	56.5	20.5	14	Left	Non-Trauma	Long	NMPK	O
F	40	1.49	45.8	20.6	12	Right	Non-Trauma	Long	NMPK	O
F	28	1.48	42.4	19.4	17	Right	Non-Trauma	Short	NMPK	O
M	26	1.69	78.2	27.4	5.5	Left	Trauma	Long	NMPK	O
M	75	1.64	52.0	19.3	25	Left	Trauma	Long	MPK	I
M	52	1.64	82.7	30.8	6.0	Right	Trauma	Medium	MPK	I
F	60	1.58	58.3	23.4	56	Right	Trauma	Short	MPK	O
M	55	1.85	70.9	20.7	5.0	Right	Non-Trauma	Long	MPK	O
F	58	1.47	47.3	21.9	27	Right	Trauma	Short	NMPK	O
F	64	1.61	46.4	17.9	18	Right	Non-Trauma	Long	MPK	I
-	48.8	1.63	59.5	22.3	19.4	-	-	-	-	-
-	14.5	0.10	12.4	3.38	13.0	-	-	-	-	-

BMI, body mass index; TSAmp, time since amputation; NMPK, non-microprocessor knee; MPK, microprocessor knee; I, inside; O, outside.

### 2.2 Experimental procedure

This experiment was conducted at both Tokyo University of Science and affiliated outdoor sports facilities. Participants were instructed to perform a FTST movement, moving from a long-sitting position to an upright standing posture at a self-selected, comfortable pace. They were permitted to employ any movement strategy required to complete the task, provided that no external assistance or environmental support (e.g., walls, furniture, or other objects) was utilized. To capture each participant's spontaneous and unprompted strategy, only a single trial was performed.

At the initiation of each trial, the examiner stood in front of the participant and provided a standardized verbal cue: “Ready, start.” To minimize experimental bias and preserve the participant's natural movement pattern, detailed task instructions were provided in advance; however, no demonstrations or prescriptive guidance regarding movement execution were given. Participants were instructed to begin from a long-sitting position and complete the task by achieving a fully upright standing posture with the arms resting naturally at the sides. This procedure was designed to elicit each participant's typical FTST behavior under self-selected conditions. The successful trial was defined as achieving an upright standing posture with the arms resting naturally at the sides.

### 2.3 Video recording setup

All FTST trials were recorded at a frame rate of 60 Hz and a spatial resolution of 2160 × 3840 pixels using a smartphone (iPhone 15 Pro Max, Apple Inc., USA) mounted on a tripod to capture a clear frontal-plane view of each participant's movement. The camera was positioned 0.7 meters anterior to the participant's toes in the long-sitting position and elevated to a height of 2.0 m. This configuration was designed to standardize the field of view and ensure adequate visibility for subsequent qualitative analysis of movement strategies.

### 2.4 Data analysis

Based on the procedures described by Iwase et al. (2011) and Ulbrich et al. (2000), three independent reviewers evaluated the movement patterns observed during the FTST task (13, 15). The three reviewers consisted of a researcher with 19 years of experience in amputee locomotion research, a physiotherapist with 14 years of experience in amputee rehabilitation and related clinical practice, and a graduate student majoring in biomechanics with three years of research experience. The number and characteristics of the FTST movement patterns were not predetermined before video analysis. Each reviewer independently examined the complete FTST movement sequence for each participant, focusing on observable movement characteristics, including trunk rotation, upper-extremity contact with the floor, lower-limb positioning, and the sequence of body support during the transition from long sitting to standing. Participants exhibiting similar movement characteristics were grouped into the same movement pattern. Following identification of the two movement patterns, their distinguishing characteristics were operationally defined based on the observed movement sequences. Pattern 1 was defined as a direct transition toward standing without adopting a quadruped-like posture, in which the intact lower limb was positioned to support the rise and the prosthetic knee remained extended; upper-limb support on the floor could be used, but bilateral hand support associated with a quadruped-like posture was absent. Pattern 2 was defined by substantial trunk and pelvic rotation from the long-sitting position, followed by bilateral hand contact with the floor and transition through a quadruped-like posture before progressing to upright standing; the prosthetic knee remained extended during the transition. The presence or absence of transition through a quadruped-like posture, together with the associated trunk rotation and bilateral hand support, served as the principal distinguishing feature between the two patterns. The recordings were reviewed on a computer monitor, allowing the reviewers to replay the footage and examine individual frames as needed to facilitate accurate classification. Inter-rater reliability for the initial independent classifications was assessed using Fleiss' kappa (*κ*), as three raters independently assigned each participant to one of two categorical movement patterns (Pattern 1 or Pattern 2). In cases of disagreement, the reviewers subsequently reviewed the video recordings together and reached a final classification by consensus.

FTST time was determined for each participant using Kinovea software (Version 0.9.5; Kinovea open-source project). Movement initiation was defined as the first frame in which observable movement of any body segment occurred from the long-sitting position. Task completion was defined as the first frame in which the participant achieved a fully upright standing posture, with both feet stationary and no further observable movement of the upper extremities. The same video recordings used for movement classification were employed for timing analysis. To ensure precise frame selection, the recordings were reviewed repeatedly on a computer monitor. For the assessment of intra-rater reliability of FTST duration, the same investigator repeated the timing analysis for a subset of eight participants (50% of the sample) without reference to the initial measurements. Intra-rater reliability between the initial and repeated measurements was assessed using a two-way mixed-effects, single-measure intraclass correlation coefficient [ICC(3,1)] with a 95% confidence interval. All temporal measurements were performed by a single investigator to maintain consistency across participants.

### 2.5 Statistical analysis

Demographic and clinical variables were compared between the two observed FTST movement pattern groups. Variables analyzed included sex, age, body height, body mass, body mass index (BMI), time since amputation, side and cause of amputation, residual limb length, type of prosthesis, and FTST time. Given the small and unequal group sizes (Pattern 1, *n* = 11; Pattern 2, *n* = 5), these between-group analyses were considered exploratory, with emphasis placed on effect-size estimates and their uncertainty rather than statistical significance alone. Residual limb length was assessed by a certified prosthetist and categorized into four groups based on the proportion of the residual femur relative to the intact side: short (≤1/3), middle (≤2/3), long (≥2/3), and knee disarticulation (KD).

Normality of continuous variables (age, height, body mass, BMI, time since amputation, and FTST time) was assessed using the Shapiro–Wilk test. Levene's test was employed to evaluate homogeneity of variances. For variables satisfying the assumptions of both normality and homogeneity of variance, unpaired Student's t-tests were used to compare the means between the two movement strategy groups. When normality was met but homogeneity was violated, Welch's *t*-tests were applied. In instances where normality was violated, the Mann–Whitney U test (or Wilcoxon rank-sum test) was used to compare distributions or medians. Categorical variables (sex, amputation side, cause of amputation, residual limb length category, and type of prosthesis) were analyzed using Fisher's exact test due to limited sample sizes.

Statistical significance was set at *p* < 0.05 for all analyses. Effect sizes were also calculated and interpreted using established benchmarks (16, 17): Hedges' g for continuous variables (small = 0.2, medium = 0.5, large = 0.8) and correlation coefficient *r* for non-parametric comparisons (small = 0.1, medium = 0.3, large = 0.5).

## 3 Results

### 3.1 Characteristics of the ftst movement patterns

Two distinct FTST strategies were observed in the present sample of 16 individuals with uTFA/KD and were categorized by three independent reviewers (Figure 1). The three reviewers reached unanimous agreement for 13 of the 16 participants (81.3%). For the remaining three participants, two reviewers classified the movement as Pattern 1 and one reviewer as Pattern 2. Overall inter-rater agreement was substantial (Fleiss' *κ* = 0.733), with an observed agreement of 87.5%. Following consensus discussion, all three discrepant cases were classified as Pattern 1, resulting in 11 participants in Pattern 1 and five in Pattern 2. As illustrated in Figure 1A, the first pattern (P1) was observed in 11 participants (68.8%). In this pattern, participants elevated their bodies using the intact limb, with assistance from one or both upper limbs. During the rising phase, the prosthetic limb remained fully extended, with the knee joint held in terminal extension. The second pattern (P2), identified in 5 participants (31.3%), was characterized by an initial rotation of the upper trunk, followed by pelvic rotation to assume a quadruped-like posture (Figure 1B). From this position, participants elevated the trunk with bilateral hand support and progressed to standing. As in pattern P1, the prosthetic knee remained extended throughout the transition. Comparative analysis of demographic characteristics across the two movement pattern groups is presented in the following section.

**Figure 1:**
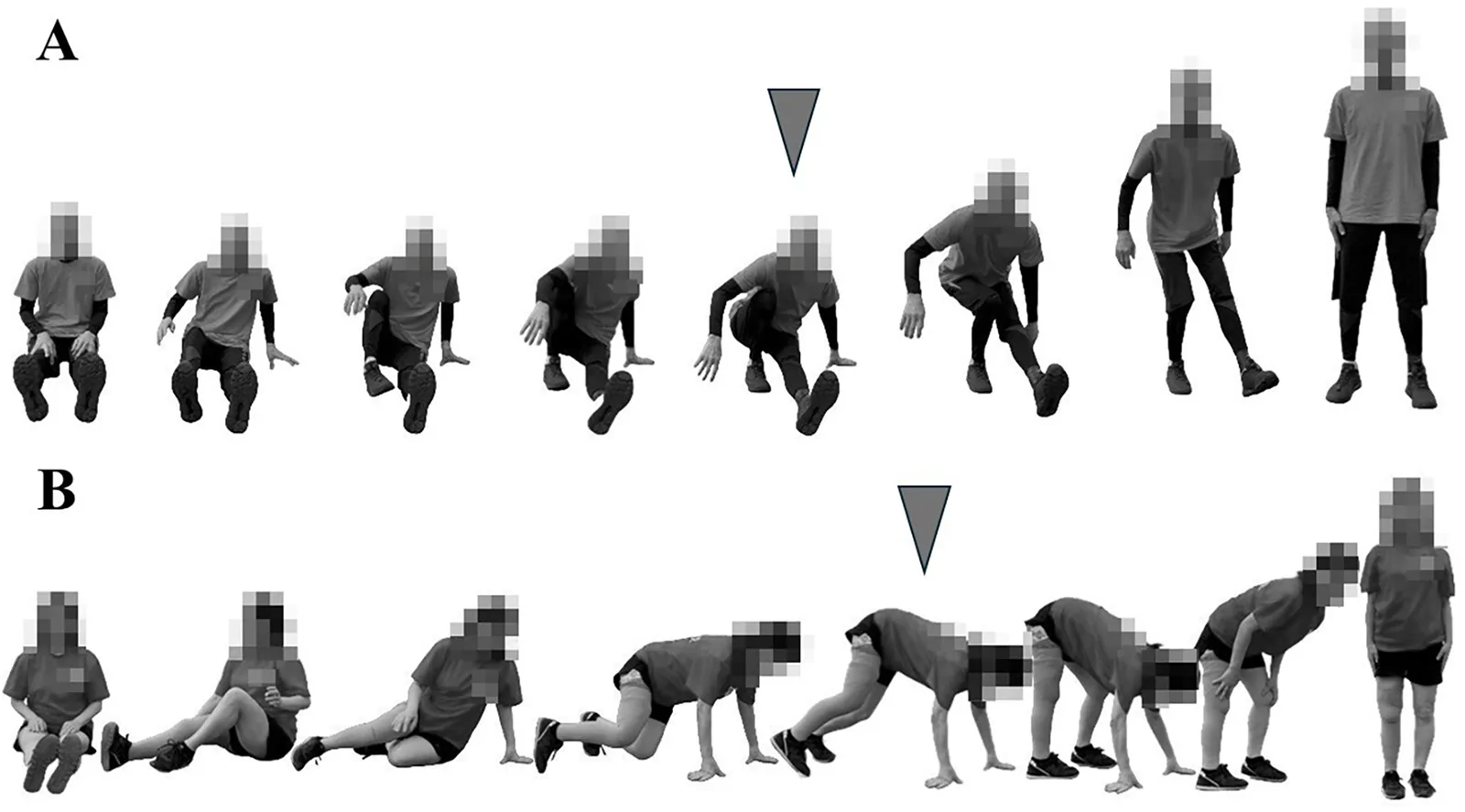
Representative image sequences illustrating two floor-to-stand transition (fTST) movement patterns identified by independent reviewers. **(A)** Pattern 1: Participants rose primarily using the intact limb (right leg), with assistance from one or both upper limbs, while the prosthetic limb (left leg) remained fully extended. **(B)** Pattern 2: Participants rotated the trunk and pelvis to assume a quadruped-like posture before progressing to standing. The prosthetic knee (right leg) remained extended throughout the transition. In both patterns, the prosthetic knee was maintained in terminal extension throughout the transition.

### 3.2 Comparisons of demographic characteristics among patterns

Exploratory comparisons of demographic and clinical characteristics between the two FTST strategy groups are presented in Table 2. Given the small and unequal group sizes (Pattern 1, *n* = 11; Pattern 2, *n* = 5), these comparisons should be interpreted cautiously. Hedges' *g* was 1.615 (95% CI: 0.400–2.830) for FTST time and 0.877 (95% CI: −0.229 to 1.983) for age, with wide confidence intervals indicating considerable uncertainty in the estimated between-group effects. Intra-rater reliability of FTST duration was excellent, with an ICC(3,1) of 0.996 (95% CI: 0.981–0.999).

**Table 2:** Demographic data of the two patterns (mean ± standard deviation).

Characteristics	Pattern 1 (N=11)	Pattern 2 (N=5)	Statistics
FTST Time (s)	4.20 (± 1.07)	7.47 (± 2.50)	Welch's *t*-test *t* = −2.336, *p* = 0.0712
Age (year)	44.7 (± 15.7)	57.8 (± 4.12)	Unpaired *t*-test *t* = −1.720, *p* = 0.1075
Height (m)	1.63[± 0.09]	1.63 (± 0.12)	Mann–Whitney U test U = 34.500, *p* = 0.4602
Mass (kg)	58.8 (± 11.5)	61.1 (± 13.99)	Unpaired *t*-test *t* = −0.331, *p* = 0.7457
BMI (kg/m*2)	22.0 [± 2.82]	22.9 (± 4.31)	Mann–Whitney U test U = 23.000, *p* = 0.6612
TSAmp (year)	18.1 (± 9.01)	22.4 (± 18.66)	Unpaired *t*-test *t* = −0.586, *p* = 0.5671
Sex			
Male	8	2	Fisher's exact test
Female	3	3	*p* = 0.2995
Amputation Side			
Right	6	5	Fisher's exact test
Left	5	0	*p* = 0.1186
Cause of Amputation			
Trauma	4	3	Fisher's exact test
Non-Trauma	7	2	*p* = 0.5962
Residual Limb Length			
Short	1	2	Fisher's exact test
Middle	2	1	*p* = 0.6538
Long	7	2	
KD	1	0	
Prosthetic Knee Unit			
MPK	5	4	Fisher's exact test
NMPK	6	1	*p* = 0.3077

## 4 Discussion

In this study, FTST movements in individuals with uTFA were classified into two distinct patterns (Figure 1). However, no significant differences were observed in demographic or clinical characteristics between the pattern groups (Table 2). These findings indicate that the two FTST strategy groups observed in the present sample were not significantly differentiated by the demographic characteristics examined. Other factors potentially associated with FTST strategy selection require further investigation. Accordingly, rehabilitation interventions aimed at improving FTST performance should prioritize movement-specific strategies and biomechanical support rather than relying solely on patient characteristics. Given the direct relevance of FTST to post-fall recovery, promoting safer and more efficient strategies for transitioning from the floor to an upright standing posture represents an important clinical objective.

Two FTST movement patterns were observed among individuals with uTFA in the present study. These observed patterns differed from those previously reported in non-amputee populations. For example, Iwase et al. (2011) identified three distinct FTST strategies from the supine position in older adults: (i) rising from a position in which both hands and feet contact the floor with both legs extended, (ii) rising from a half-kneeling position, and (iii) rising from a squatting position (13). Notably, the half-kneeling strategy, which has been observed in individuals with TTA (12), was not observed in the present uTFA cohort. This difference may reflect biomechanical, prosthesis-related constraints specific to transfemoral amputation, including the use of microprocessor or non-microprocessor knees and their operating or locking behavior during the FTST, as well as prosthetic foot characteristics, socket design and comfort, residual limb characteristics, kneeling tolerance, previous training or experience, and individual preference (18). Although prosthetic knee type was included among the participant characteristics in the present study, the sample size was insufficient to examine the effects of specific prosthetic components or configurations on FTST strategy selection. Furthermore, previous studies reported that individuals with uTFA may be at greater risk of falls and fall-related injuries than those with transtibial amputation (2, 3). Fear of falling and reduced balance confidence have also been associated with mobility limitations in individuals with lower-limb amputation (19). Therefore, these psychological factors may influence the selection of FTST strategies, particularly when transitioning through positions that impose greater demands on postural stability. However, fear of falling and balance confidence were not assessed in the present study. The biomechanical and prosthesis-related constraints associated with transfemoral amputation may make transitions through a half-kneeling position less feasible, while the potential contribution of psychological factors warrants further investigation.

No statistically significant differences were observed between the two FTST patterns in any of the examined continuous or categorical variables (Table 2). Although relatively large Hedges' *g* values were observed for age (*g* = 0.877, 95% CI: −0.229 to 1.983) and FTST time (*g* = 1.615, 95% CI: 0.400–2.830), the wide confidence intervals indicate considerable uncertainty in the magnitude of these between-group differences. Nevertheless, the observed tendencies are generally consistent with previous findings. Iwase et al. (2011) reported that individuals who performed the FTST from a position involving both hands and feet in contact with the floor while both legs remained extended required more time to complete the task than those who used a squatting strategy (13). Similarly, Burton et al. (2023) reported that increasing age was associated with greater use of movement strategies involving trunk rotation and longer FTST times (20). In the present sample, participants who adopted the trunk-rotation strategy (P2) also tended to be older and required more time to complete the FTST than those who adopted P1; however, these between-group differences did not reach statistical significance. The factors underlying these observed tendencies remain unclear, as potentially relevant factors, such as muscle strength and balance control, were not assessed in the present study. Further studies with larger samples and direct assessment of these factors are required to determine their potential associations with FTST strategy selection.

Several limitations of the present study should be acknowledged. First, the initial posture may have influenced the FTST strategies observed. Participants began the task from a standardized long-sitting position, which does not fully replicate real-world post-fall scenarios. Given the lack of an established experimental protocol in FTST research, initiating the task from alternative positions—such as supine or prone—may have elicited different movement strategies. Additionally, participants performed the FTST task without the use of assistive devices (e.g., chairs or handrails). Although all individuals were able to complete the task independently, it is plausible that assistive device use in daily life may lead to alternative FTST strategies. In addition, movement strategies were evaluated using video recordings obtained from a single frontal camera. Although this view allowed identification of the principal features used to distinguish the observed FTST strategies, movements occurring outside the frontal plane, particularly trunk and pelvic rotation, may not have been fully captured. Therefore, some movement characteristics may have been overlooked or incompletely characterized. Future studies incorporating multiple camera views or three-dimensional motion analysis would enable a more comprehensive assessment of FTST movement strategies. Second, only a single FTST trial was performed for each participant. Although this approach was intended to capture the participant's spontaneous movement strategy without potential adaptation associated with repeated task performance, FTST strategies may vary across repeated attempts. Therefore, the present findings cannot establish whether the observed strategy represents a stable movement pattern for each participant or the strategy adopted during that specific trial. Future studies should include multiple trials to assess within-participant consistency and the reproducibility of FTST strategies in individuals with uTFA. Additionally, task instructions may have influenced movement selection. Participants were instructed to perform the transition at a self-selected, comfortable pace. However, different task demands—such as rising as quickly as possible—may have produced different strategy selections. Future studies should incorporate phase-based analyses of the FTST to characterize differences in movement progression within and between individuals with uTFA. Third, variability in participants' rehabilitation environments may have affected the development of FTST strategies. The study did not document the setting (e.g., inpatient, outpatient, home-based) or content and time of participants' rehabilitation programs. Previous research suggests that rehabilitation context and intensity can significantly influence functional outcomes. For example, past findings reported that both the setting and quality of rehabilitation impact recovery of physical function and independence (20, 21). Optimizing the rehabilitation environment and promoting early, structured intervention should therefore remain clinical priorities. Finally, the sample size was relatively small, which limits the generalizability of the findings and reduces the precision of the estimated between-group effects. In particular, the wide 95% confidence intervals for Hedges' *g* indicate considerable uncertainty regarding the magnitude of the observed effect sizes. Further, the present sample included one participant with knee disarticulation, whereas the remaining 15 participants had transfemoral amputation. Knee disarticulation is anatomically distinct from transfemoral amputation and may confer different biomechanical and prosthetic characteristics relevant to the FTST. Because only one participant with knee disarticulation was included, its specific influence on FTST strategy could not be evaluated. Furthermore, participants may not have been consciously aware of how they typically perform the FTST in daily life, potentially affecting the ecological validity of the observed strategies. Previous research has reported that approximately 53% of individuals with LLA were unable to perform the FTST independently. In contrast, the present study included, as an inclusion criterion, only individuals with uTFA/KD who were able to safely and independently complete the FTST. Moreover, participants were identified through targeted recruitment by collaborating certified prosthetists, and complete recruitment-flow information, including the numbers of individuals considered for participation, unable to independently perform the FTST, or declining participation, was not available. Consequently, potential selection bias associated with the recruitment procedure cannot be fully evaluated. Therefore, the present findings represent FTST strategies adopted by a relatively high-functioning subgroup and should not be generalized to the broader population, particularly individuals who require assistance to rise from the floor. Future studies should include larger sample sizes, adopt standardized experimental protocols, and obtain detailed participant histories, including previous rehabilitation or FTST training, fall history, duration of current prosthesis use, and functional or physical activity level. Additionally, detailed prosthetic characteristics, including knee type and operating mode, prosthetic foot characteristics, and socket design and comfort, as well as fear of falling and balance confidence, should also be assessed to determine their potential associations with FTST strategy selection.

## 5 Conclusion

The present study identified two distinct FTST strategies observed in the current sample of individuals with uTFA/KD: a rise primarily supported by the intact limb with upper-limb assistance and a trunk-rotation strategy involving transition through a quadruped-like posture. No statistically significant differences in the examined demographic characteristics were observed between the two strategy groups, and the factors associated with FTST strategy selection remain unclear. The findings may provide useful information for developing movement-specific rehabilitation approaches aimed at improving safety and efficiency during post-fall recovery. However, given the relatively small sample size, these two strategies should be regarded as movement patterns observed in the present cohort rather than an exhaustive categorization of the observed FTST strategies in the broader uTFA population. Further studies with larger samples and repeated FTST trials are required to determine the range and consistency of FTST strategies among individuals with uTFA and to identify factors associated with strategy selection.

## Data Availability

The data that support the findings of this study are available from the corresponding author upon reasonable request.
